# Diagnostic value of metagenomic next-generation sequencing in patients with febrile lung cancer with negative conventional microbiological tests and without neutropenia

**DOI:** 10.3389/fcimb.2026.1715563

**Published:** 2026-03-13

**Authors:** Changjian Wang, Mengyun Min, Zhendong Dai, Geng Wang, Yangchenxi Wang, Ting Hu, Yuxi Ma, Sheng Zhang, Chuangyan Wu, Rui Zhou

**Affiliations:** 1 Department of Thoracic Surgery, Union Hospital, Tongji Medical College, Huazhong University of Science and Technology, Wuhan, China; 2 Department of Oncology, The Second People’s Hospital of China Three Gorges University, Yichang, China; 3 Department of Gastrointestinal Surgery, Union Hospital, Tongji Medical College, Huazhong University of Science and Technology, Wuhan, China; 4 Cancer Center, Union Hospital, Tongji Medical College, Huazhong University of Science and Technology, Wuhan, China; 5 Institute of Radiation Oncology, Union Hospital, Tongji Medical College, Huazhong University of Science and Technology, Wuhan, China; 6 Hubei Key Laboratory of Precision Radiation Oncology, Wuhan, China

**Keywords:** blood culture, diagnosis, infectious fever, lung cancer, metagenomic next-generation sequencing (mNGS), non-neutropenic

## Abstract

**Introduction:**

Fever in nonneutropenic lung cancer often remains microbiologically unresolved because of the limitations of conventional microbiological tests (CMT). We assessed whether plasma metagenomic next-generation sequencing (mNGS) improves diagnostic yield and accelerates defervescence in these patients.

**Methods:**

We retrospectively analyzed 53 CMT-negative febrile lung cancer patients (August 2023–October 2024). Patients were classified into high-suspicion infectious fever (HSIF) or high-suspicion tumor fever (HSTF) groups based on mNGS results, and clinical management was adjusted accordingly.

**Results:**

mNGS identified pathogens in 69.8% (37/53) of patients, commonly including *Epstein–Barr virus*, *Mycobacterium tuberculosis*, and *Candida albicans*. Patients in the HSIF group showed significantly higher baseline inflammatory markers than those in the HSTF group. Importantly, following mNGS-guided antimicrobial therapy, the HSIF group achieved significantly higher defervescence rates at 48 h (73.0% *vs*. 37.5%; *p* = 0.029) and 96 h (89.2% *vs*. 68.8%; *p* = 0.027) compared to the HSTF group.

**Discussion:**

In conclusion, in CMT-negative, nonneutropenic febrile lung cancer, plasma mNGS significantly increases pathogen detection and informs antimicrobial decisions associated with earlier defervescence, although interpretation is limited by the retrospective design and lack of an independent gold standard.

## 1 Introduction

Lung cancer is one of the most common and lethal malignancies worldwide and accounts for a major public health challenge (Sung et al., 2021). Patients with lung cancer are highly susceptible to infectious complications due to airway obstruction, treatment-related mucosal injury, and systemic immune dysfunction, which together contribute to poor outcomes (Rosolem et al., 2012; Rolston, 2017). Although neutropenia is a well-recognized risk factor for infection (Reyna-Figueroa et al., 2015), fever is also common in patients with nonneutropenic lung cancer, and the differential diagnoses include bacterial infection and tumor fever (TF) (Shomali et al., 2012). Fever is often the earliest and sometimes only clinical indicator of infection (Akinosoglou et al., 2013). It is generally considered a protective host immune response against invading pathogens (Marik, 2000). Fever in lung cancer may also be tumor-related and diagnosed by exclusion (Zell and Chang, 2005). Insufficient early discrimination can expose nonbacterial episodes to unnecessary antibiotics with attendant toxicity and resistance, whereas the true infection risk delays definitive therapy and worsens outcomes (Zhao et al., 2018; Messacar et al., 2017).

Conventional microbiological techniques—cultures and serology—are foundational yet limited by modest sensitivity, prolonged turnaround, and narrow pathogen coverage, particularly after prior antibiotic exposure (Goldberg et al., 2015; Grumaz et al., 2016). Performance further depends on sampling timing, technique, and organism growth requirements, leading to missed fastidious or slow-growing pathogens and diagnostic delays (Gu et al., 2019). Traditional assays also cannot profile the full microbial community, reducing utility in polymicrobial or otherwise complex infections (Wang et al., 2019). These constraints frequently yield CMT-negative evaluations in nonneutropenic patients, prolonging empiric, trial-and-error management.

Metagenomic next-generation sequencing (mNGS) is an advanced diagnostic technology capable of simultaneously detecting a broad spectrum of pathogens, including bacteria, viruses, fungi, and parasites (Tang et al., 2021). Unlike conventional culture-based methods, mNGS enables the comprehensive analysis of nucleic acids present in clinical specimens, most commonly blood, using high-throughput sequencing platforms. Human-derived sequences are bioinformatically removed, and the remaining microbial reads are aligned to comprehensive reference databases for precise taxonomic classification (Wooley et al., 2010). This approach allows the simultaneous detection of diverse pathogens from a single clinical sample (Rodino and Simner, 2024). As mNGS is not dependent on organism viability, it retains value despite antecedent antibiotics and is particularly useful for mixed, fastidious, or rare infections (Diao et al., 2022; Han et al., 2019). For example, Lin et al. (2022) reported that mNGS applied to bronchoalveolar lavage fluid significantly improved pathogen detection compared with conventional methods, particularly for *Pneumocystis jirovecii* and co-infections in immunocompromised patients. In addition to its broad detection capability, mNGS provides semiquantitative data that can be used to estimate pathogen loads and identify antimicrobial resistance genes, offering actionable insights for precision antimicrobial therapy (Sahoo et al., 2013; Salipante et al., 2014).

Against this backdrop, the present study aimed to evaluate plasma-based mNGS in patients with febrile lung cancer who are nonneutropenic and CMT-negative, specifically to determine whether it provides incremental diagnostic yield, thereby supporting immediate, result-guided antimicrobial decisions, which are associated with earlier defervescence.

## 2 Materials and methods

### 2.1 Patients and fever episode definition

**Figure 1:**
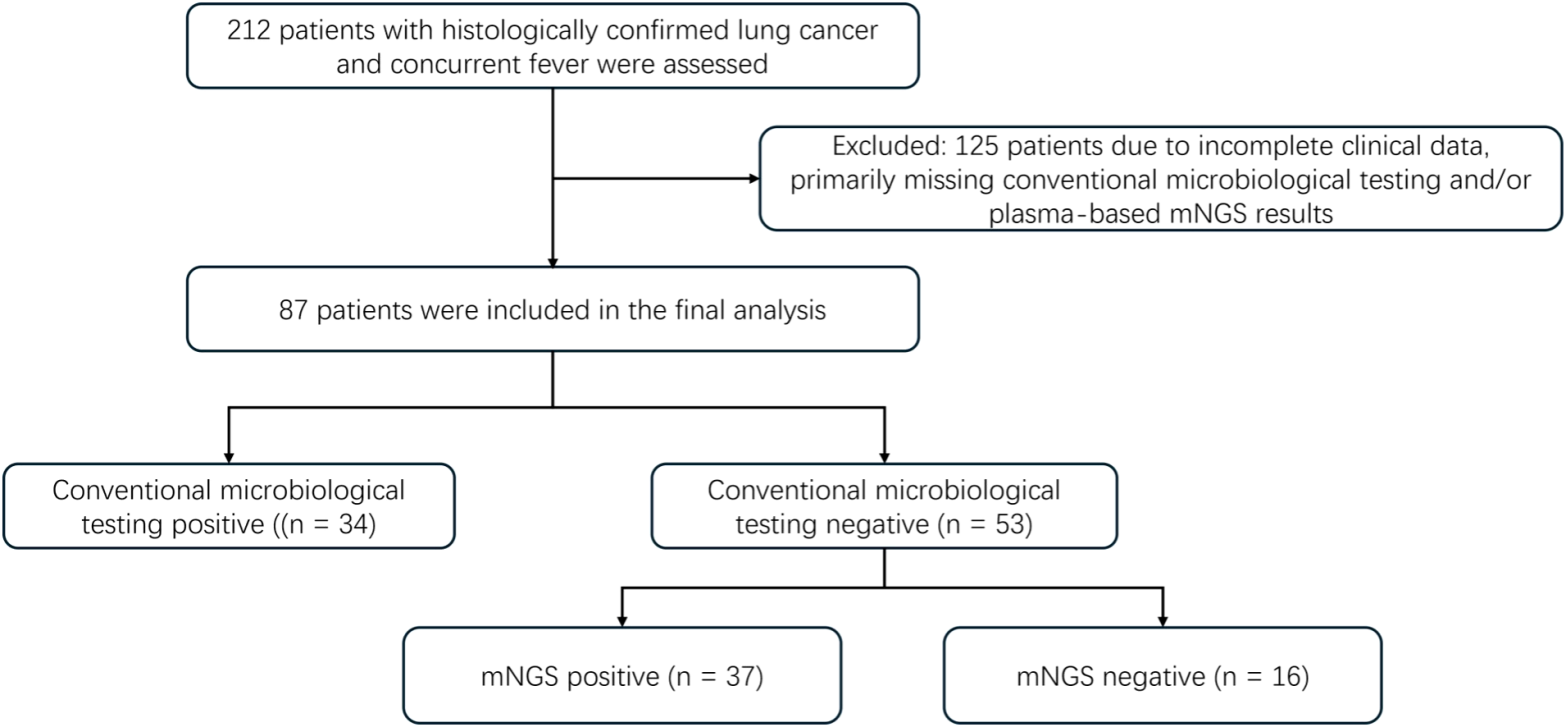
Flowchart of patient selection and subgroup classification.

This single-center, retrospective cohort study was conducted at the Cancer Center of the Union Hospital, Tongji Medical College, Huazhong University of Science and Technology (August 2023–October 2024). Consecutive adults (age ≥ 18 years) with histologically confirmed lung cancer who developed an eligible fever episode at admission or during hospitalization were screened. In total, 212 patients met the screening criteria; after excluding 125 patients for prespecified reasons, most commonly incomplete conventional microbiology and/or plasma mNGS data, 87 patients remained for analysis (**Figure 1**). Of these, 34 were positive for conventional microbiological tests (CMT) and were summarized descriptively, whereas 53 were CMT-negative and constituted the primary analysis set. The latter were stratified *a priori* based on the mNGS result into two exploratory arms: mNGS-positive high-suspicion infectious fever (mNGS-HSIF) (*n* = 37) and mNGS-negative high-suspicion tumor fever (mNGS-HSTF) (*n* = 16), forming the basis for analyses of incremental diagnostic yield, mNGS-prompted management changes, and landmark defervescence endpoints.

An eligible fever episode was defined as an axillary temperature ≥ 38.5 °C confirmed on repeat measurement within 6 h under routine care. Episodes that resolved within 12 h without systemic signs were considered transient and were not eligible. Patients with fever clearly attributable to noninfectious triggers (e.g., infusion/transfusion reactions, colony-stimulating factor use, or immediate postprocedural/anesthesia responses) were not considered eligible. Only the first eligible episode was analyzed in patients with multiple febrile events. Nonneutropenia was required; neutropenia was an exclusion criterion defined as an absolute neutrophil count (ANC) < 0.5 × 10^9^/L based on the closest complete blood count around fever onset.

The index time point (0) for outcome assessment was prespecified as the time the mNGS report became available in the electronic medical record. Per institutional practice during the study period, clinicians immediately adjusted or initiated targeted anti-infective therapy at *t*_0_ for mNGS-positive cases, whereas mNGS-negative cases generally continued oncological management and supportive care; conventional antipyretics were permitted at clinicians’ discretion in both groups.

The primary endpoint was time to defervescence from *t*_0_, defined as an axillary temperature < 37.3 °C sustained for ≥ 24 h; because antipyretics were permitted per routine care, defervescence should be interpreted as symptom control rather than definitive pathogen eradication.

Prespecified secondary summaries were defervescence proportions at 24/48/72/96 h post-*t*_0_.

### 2.2 Inclusion and exclusion criteria

#### 2.2.1 Inclusion

Patients were eligible if they met all of the following: (1) age ≥ 18 years; (2) histologically confirmed lung cancer with complete oncological records; (3) at least one eligible fever episode as defined in Section 2.1; (4) completion of blood cultures within ≤ 24 h, pathogen serology per institutional practice, and plasma mNGS within ≤ 72 h of the first eligible fever; and (5) availability of clinical data for outcome assessment through hospital discharge or day 28, whichever occurred first.

#### 2.2.2 Exclusion (operational windows)

Patients were excluded for the following reasons: (1) neutropenia, defined as ANC < 0.5 × 10^9^/L within − 24 to + 24 h of fever onset; (2) prespecified noninfectious triggers, namely, infusion-related reactions to cytotoxic/immune therapies or monoclonal antibodies during infusion or within 24 h after completion; transfusion-related reactions during or within 4–24 h after blood product administration; colony-stimulating factor-related fever within 72 h of granulocyte colony-stimulating factor (G-/GM-CSF) administration; and immediate postprocedural/anesthesia fever within 48 h of an invasive procedure or anesthesia; (3) transfer before completion of the diagnostic work-up; (4) incomplete clinical information or failed sample quality control for blood cultures, serology, or mNGS; and (5) recurrent febrile episodes in the same patient (only the first eligible episode was analyzed).

#### 2.2.3 Sampling windows and prior antimicrobial exposure

Blood cultures and pathogen serology were performed within 24 h, and plasma mNGS was performed within 72 h of fever onset.

### 2.3 Sample collection

Blood cultures and pathogen serology were performed within 24 h of the first eligible fever. Plasma for mNGS (5–10 mL of ethylenediaminetetraacetic acid [EDTA] whole blood) was obtained within 72 h of fever onset in routine practice. The exact sampling timestamps were recorded in electronic medical records, and the specimens were transported immediately to the laboratories according to institutional standard operating procedures.

### 2.4 Metagenomic next-generation sequencing

Plasma mNGS was performed using a standardized shotgun workflow (PMseq^®^; BGI, Shenzhen, China) on Illumina or MGISEQ platforms (Miao et al., 2018). Cell-free DNA was extracted from EDTA plasma, and human reads were bioinformatically removed before taxonomic classification against a curated microbial reference database (PMDB) (~ 17,500 pathogenic taxa, vendor-maintained) (Li and Durbin, 2009). Each sequencing batch incorporated an internal spike-in control, together with batch-matched negative and positive controls, to monitor potential contamination and process integrity. Library preparation followed clinical-grade QC gates (e.g., input concentration/purity, fragment size distribution, run yield, and base-calling quality with Q20/Q30 reporting). Per laboratory Standard Operating Procedures (SOP), we targeted a per-sample sequencing depth of ≥ 20 million reads to ensure analytical sensitivity.

Taxa were reported only when (i) prespecified species-level read count/RPM thresholds were met and (ii) statistical enrichment over batch-matched negative controls was achieved after host-read subtraction and background filtering. Adjudication further considered clinical plausibility and treatment response, as detailed previously (Miao et al., 2018). Low-level herpes virus detection was performed using sensitivity analyses (inclusive *vs*. strict exclusion). Turnaround time was defined as the time from laboratory receipt to report availability in the medical record and is reported as median (interquartile range [IQR]). Detailed QC metrics, database version/release data, and positivity thresholds are provided in **Supplementary Methods**.

### 2.5 Conventional microbiological testing

Blood cultures were obtained under aseptic conditions and processed in automated incubators according to the institutional SOPs. Positive bottles were subjected to Gram staining, biochemical or matrix-assisted laser desorption/ionization–time-of-flight identification, and antimicrobial susceptibility testing, as indicated, according to Clinical and Laboratory Standards Institute (CLSI) guidelines (Clinical and Laboratory Standards Institute, 2023). Pathogen serology (atypical bacteria and respiratory viruses) was performed using enzyme-linked immunosorbent assay, according to the manufacturer’s instructions, targeting *Mycoplasma pneumoniae*, *Chlamydia pneumoniae*, respiratory syncytial virus, adenovirus, and Coxsackie B virus. Results above the assay-specific cutoffs were considered positive.

### 2.6 Ethics

This single-center retrospective chart review complied with the Declaration of Helsinki and applicable regulations. The study protocol was reviewed and approved prior to any data extraction or analysis by the Institutional Review Board (IRB) of Union Hospital, Tongji Medical College, Huazhong University of Science and Technology (Approval No. 2025(0436); 06 May 2025). In line with institutional policy for minimal-risk retrospective studies using pre-existing records, the IRB granted a waiver of written informed consent. No study-specific interventions or additional specimen collection were undertaken; all tests—including plasma mNGS—were ordered as part of routine clinical care. All analyses were conducted on de-identified data only, and no patient contact occurred.

### 2.7 Statistical analyses

All analyses were performed using SPSS version 27.0 (IBM, Armonk, NY, USA), and figures were created using GraphPad Prism version 10.0 (GraphPad Software, La Jolla, CA, USA). The primary analysis set comprised CMT-negative patients classified as having mNGS-HSIF (*n* = 37) or mNGS-HSTF (*n* = 16). Continuous variables are summarized as means ± standard deviations or medians (IQRs) according to the Shapiro–Wilk assessment of normality; categorical variables are summarized as *n* (%). Statistical significance was set at *p* < 0.05.

**Figure 2:**
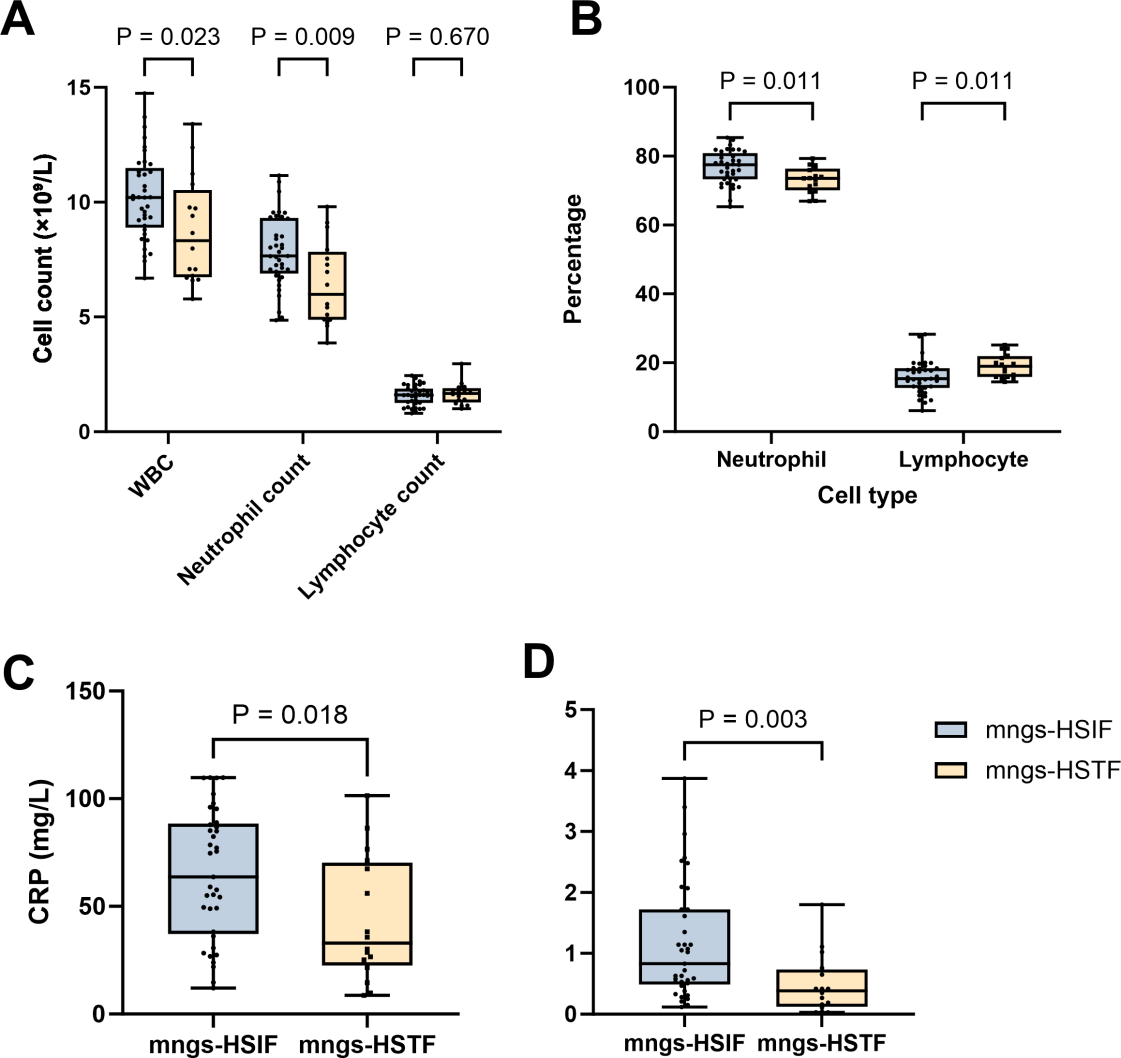
Leukocyte and inflammatory indices at fever onset by the mNGS group. **(A)** Absolute leukocyte counts—WBC, neutrophils, and lymphocytes (× 10^9^/L). **(B)** Differential counts—neutrophils and lymphocytes (%). **(C)** C-reactive protein (CRP, mg/L). **(D)** Procalcitonin (PCT, ng/mL). Boxes indicate medians with IQRs; whiskers show ranges; dots represent individual patients. Groups: mNGS-HSIF (blue) and mNGS-HSTF (beige); sample sizes: HSIF *n* = 37, HSTF *n* = 16. *p*-values are from two-sided Mann–Whitney *U* tests; ns, not significant. Exact *p*-values are reported in **Table 3**.

Baseline characteristics are descriptively presented in **Table 1** without formal hypothesis testing. Pathogen detection among mNGS-positive cases is summarized in **Table 2** as counts based on pathogen and organism type. Laboratory indices at fever onset (**Table 3**, **Figure 2**) were compared between groups using the Mann–Whitney *U* test for continuous variables and *χ*^2^ or Fisher’s exact tests for categorical variables.

**Table 1:** Baseline clinical characteristics (*n* = 53).

Clinical characteristics	Number (*n* = 53)	Percentage (%)
Sex		
Male	47	88.7
Female	6	11.3
Age (years; mean ± SD)	64.1 ± 6.3	
Smoking history		
Current	18	34.0
Previous	18	34.0
Never	17	32.1
Pathological type		
Adenocarcinoma	27	50.9
Squamous cell carcinoma	21	39.6
Small-cell lung cancer	4	7.5
Others	1	1.9
Initial cancer stage (AJCC Eighth)		
Stages I–II	3	5.7
Stage III	25	47.2
Stage IV	25	47.2
Comorbidities		
Chronic obstructive pulmonary disease (COPD)	19	35.8
Hypertension	20	37.7
Diabetes mellitus	23	43.4
Recent chemo-/radiotherapy	23	43.4

Data are presented as *n* (%), unless otherwise specified; age is presented as means ± SDs. Percentages were calculated for the primary cohort (*n* = 53). Smoking categories are mutually exclusive. Cancer stage is summarized per the AJCC Eighth edition.

**Table 2:** Pathogen detection in mNGS-positive patients.

Pathogen type	Organism	Number of detections
Bacteria	MTB	4
Bacteria	*Nocardia* spp.	3
Bacteria	*Legionella pneumophila*	2
Bacteria	*Acinetobacter baumannii*	2
Bacteria	*Klebsiella pneumoniae*	1
Bacteria	*Pseudomonas aeruginosa*	1
Bacteria	*Streptococcus pneumoniae*	1
Bacteria	*Haemophilus influenzae*	1
Bacteria	*Escherichia coli*	1
Bacteria	*Staphylococcus aureus*	1
Bacteria	*Staphylococcus epidermidis*	1
Virus	EBV	6
Virus	CMV	5
Virus	Adenovirus	5
Virus	HSV	3
Virus	Circovirus	3
Virus	JC polyomavirus	2
Virus	Human herpesvirus 7	2
Virus	Human herpesvirus 6	1
Virus	Varicella-zoster virus	1
Fungus	*Candida albicans*	4
Fungus	*Aspergillus* spp.	3
Fungus	*Pneumocystis jirovecii*	1

**Table 3:** Baseline characteristics at fever onset in HSIF versus HSTF.

Variable	mNGS-HSIF (*n* = 37)	mNGS-HSTF (*n* = 16)	*p*-value
Demographics			
Age (years)	62.00 (59.50–67.00)	66.00 (59.00–71.75)	0.613
Sex: female (*n*, %)	4/37 (10.8%)	2/16 (12.5%)	1.000
Comorbidities and habits			
Diabetes (*n*, %)	16/37 (43.2%)	7/16 (43.8%)	1.000
Hypertension (*n*, %)	14/37 (37.8%)	6/16 (37.5%)	1.000
COPD (*n*, %)	16/37 (43.2%)	3/16 (18.8%)	0.123
Smoking status (*n*, %—overall *χ*^2^*p*)			
Current	12/37 (32.4%)	6/16 (37.5%)	–
Never	10/37 (27.0%)	7/16 (43.8%)	–
Previous	15/37 (40.5%)	3/16 (18.8%)	–
Cancer and treatment			
Initial cancer stage (AJCC Eighth) (*n*, %—overall *χ*^2^*p*)			
Stages I–II	2/37 (5.4%)	1/16 (6.2%)	–
Stage III	17/37 (45.9%)	8/16 (50.0%)	–
Stage IV (metastatic)	18/37 (48.6%)	7/16 (43.8%)	–
Recent chemo-/radiotherapy (*n*, %)	17/37 (45.9%)	6/16 (37.5%)	0.764
Laboratory at fever onset			
Temperature (*T*_max_ within 24 h, °C)	39.10 (38.50–40.10)	38.90(38.60–39.60)	0.365
WBC (× 10^9^/L)	10.20 (6.69–14.74)	8.32(5.78–13.40)	0.023
CRP (mg/L)	63.67 (12.06–109.84)	32.91 (8.74–101.45)	0.018
PCT (ng/mL)	0.83 (0.12–3.87)	0.38 (0.03–1.80)	0.003
ESR (mm/h)	35.10 (13.60–83.70)	27.90 (10.20–58.50)	0.143
Neutrophil count (× 10^9^/L)	7.66 (4.86–11.16)	5.98 (3.87–9.80)	0.009
Neutrophil percentage (%)	77.48 (65.33–85.42)	73.58 (66.96–79.34)	0.011
Lymphocyte count (× 10^9^/L)	1.61 (0.81–2.45)	1.67 (1.01–2.96)	0.670
Lymphocyte percentage (%)	15.37 (6.07–28.30)	18.97 (14.47–25.18)	0.011

Data are presented as medians (IQRs), unless otherwise specified. *p*-values are from the Mann–Whitney *U* test (continuous variables) and *χ*^2^ or Fisher’s exact test (categorical variables).

*T_max_*, maximum temperature within 24 h; *HSIF*, high-suspicion infectious fever; *HSTF*, high-suspicion tumor fever; *COPD*, chronic obstructive pulmonary disease; *WBC*, white blood cell; *CRP*, C-reactive protein; *PCT*, procalcitonin; *ESR*, erythrocyte sedimentation rate.

**Figure 3:**
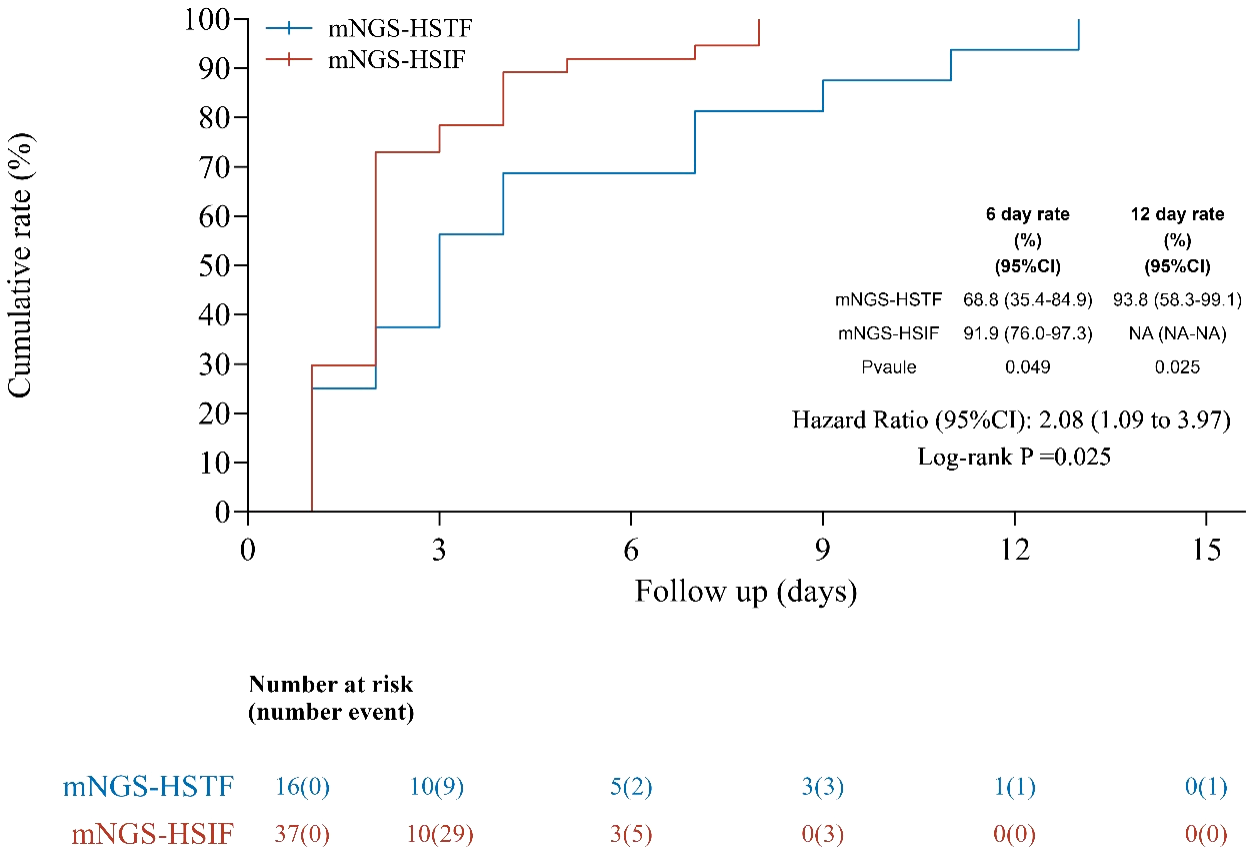
Kaplan–Meier estimates of time to defervescence from *t*_0_ in the mNGS group. The event was defined as the first time after *t*_0_ (mNGS report availability) at which all temperature measurements over the subsequent 24-h window were < 37.3 °C. Patients without defervescence were right-censored at discharge or day 28; tick marks indicate censoring. The curve displays the cumulative probability of defervescence (1−*S*[*t*]) with the number at risk below the *x*-axis. Groups were compared using the log-rank test; univariate Cox models provided hazard ratios with 95% CIs.

The primary endpoint was defervescence, defined *a priori* as axillary temperature < 37.3 °C sustained for ≥ 24 h. Landmark proportions of patients defervesced by 24/48/72/96 h after *t*_0_ (time of mNGS report availability) were compared with Fisher’s exact tests and exact 95% confidence intervals (CIs) (**Table 4**). For time-to-event analysis (**Figure 3**), time to defervescence was measured from *t*_0_ to the first time point at which all subsequent temperature readings over a continuous 24-h window were < 37.3 °C. Patients without defervescence were right-censored at hospital discharge or day 28, whichever occurred first. Unadjusted group differences were evaluated using the log-rank test, and univariate Cox models provided hazard ratios (HRs) with 95% CIs (Efron method for ties). No multivariate modeling or multiple testing adjustment (e.g., false discovery rate) was performed. Given the exploratory nature of this study, no multiplicity adjustment was applied, and the *p*-values were descriptive. Interpretation prioritizes effect sizes and 95% CIs (e.g., median differences and risk differences). Missing patterns were reviewed, and complete case analyses were performed.

**Table 4:** Landmark defervescence proportions.

Timepoints	HSIF *n*/*N*	HSIF % (95% CI)	HSTF *n*/*N*	HSTF % (95% CI)	*p*-value (Fisher)
24 h	11/37	29.7% (15.9–47.0)	4/16	25.0% (7.3–52.4)	1.000
48 h	27/37	73.0% (55.9–86.2)	6/16	37.5% (15.2–64.6)	0.029
72 h	29/37	78.4% (61.8–90.2)	9/16	56.3% (29.9–80.2)	0.182
96 h	33/37	89.2% (74.6–97.0)	11/16	68.8% (41.3–89.0)	0.027

Defervescence was defined as axillary temperature < 37.3 °C sustained for ≥ 24 h. Timepoints were measured from *t*_0_ (mNGS report availability). *p*-values were obtained from a two-sided Fisher’s exact test.

*HSIF*, mNGS-positive high-suspicion infectious fever; *HSTF*, mNGS-negative high-suspicion tumor fever.

## 3 Results

### 3.1 Overview of the study population

The baseline features of the 53 CMT-negative patients are summarized in **Table 1**. The cohort was predominantly composed of men (88.7%), with a mean age of 64.1 years ± 6.3 years. Non-small cell lung cancer was common, with adenocarcinoma accounting for 50.9% (27/53) and squamous cell carcinoma accounting for 39.6% (21/53) (combined 90.5%), whereas small cell lung cancer and other histologies accounted for 7.5% and 1.9%, respectively. Most patients had advanced disease (stages III–IV, 94.4%). Ever-smoking was reported by 68.0% of the patients, and diabetes (43.4%), hypertension (37.7%), and chronic obstructive pulmonary disease (35.8%) were frequent. Recent chemo-/radiotherapy was documented in 43.4% of the patients.

### 3.2 Diagnostic yield of mNGS

**Figure 4:**
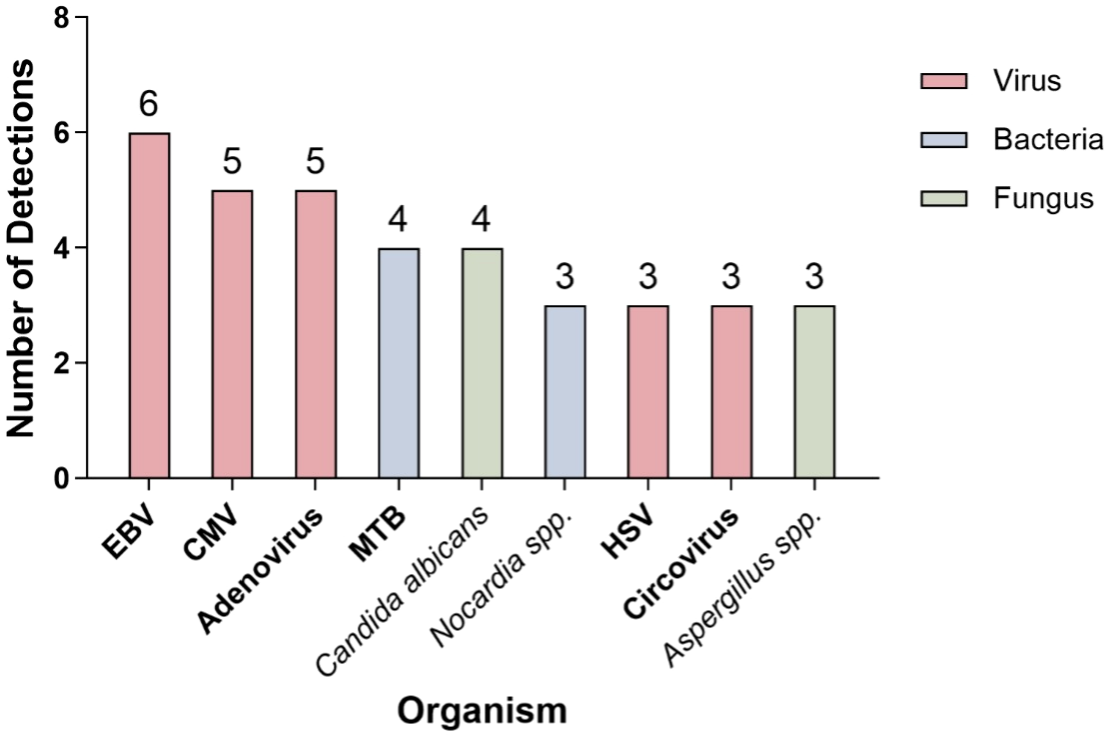
Distribution of the most frequently detected pathogens in the mNGS-positive group. Pathogens were color-coded by type: viruses (red), bacteria (blue), and fungi (green). Abbreviations are provided in the legend: EBV, Epstein–Barr virus; CMV, cytomegalovirus; HSV, herpes simplex virus; MTB, *Mycobacterium tuberculosis*.

Among the 53 CMT-negative patients, plasma mNGS identified ≥ 1 pathogen in 37 (69.8%) patients, whereas 16 (30.2%) were mNGS-negative. Across the 37 mNGS-positive cases, 54 pathogen detections were recorded: 18 bacterial, 28 viral, and eight fungal detections. The most frequent bacteria were *Mycobacterium tuberculosis* (*n* = 4) and *Nocardia* spp. (*n* = 3), whereas the leading fungi were *Candida albicans* (*n* = 4) and *Aspergillus* spp. (*n* = 3). The viral spectrum was dominated by herpes viruses, notably the *Epstein–Barr virus* (*n* = 6), *cytomegalovirus* (*n* = 5), and *adenovirus* (*n* = 5). Full details are provided in **Table 2**, and the distribution of the most frequently detected pathogens is illustrated in **Figure 4**.

### 3.3 Clinical characteristics of mNGS-high-suspicion tumor fever and mNGS-HSTF

The baseline demographics and oncological variables were comparable across the groups (**Table 3**). At fever onset, mNGS-HSIF showed higher inflammatory indices than mNGS-HSTF, including white blood cell count (median, 10.20 *vs*. 8.32 × 10^9^/L; *p* = 0.023), neutrophil count (7.66 *vs*. 5.98 × 10^9^/L, *p* = 0.009), C-reactive protein (CRP) (63.67 mg/L *vs*. 32.91 mg/L, *p* = 0.018), and procalcitonin (PCT) (0.83 ng/mL *vs*. 0.38 ng/mL, *p* = 0.003) (**Figures 2A, C, D**). Neutrophil and lymphocyte percentages also differed (77.48% *vs*. 73.58%, *p* = 0.011 and 15.37% *vs*. 18.97%, *p* = 0.011, respectively; **Figure 2B**), whereas lymphocyte count (1.61 *vs*. 1.67 × 10^9^/L; *p* = 0.670), erythrocyte sedimentation rate (35.10 mm/h *vs*. 27.90 mm/h, *p* = 0.143), and peak temperature (39.10 °C *vs*. 38.90 °C, *p* = 0.365) were similar. Full statistics are provided in **Table 3**.

### 3.4 Treatment response and clinical outcomes

In accordance with institutional practice, the receipt of an mNGS-positive report prompted immediate result-guided antimicrobial adjustments in the HSIF arm, whereas mNGS-negative cases (HSTF) generally continued oncological treatment with supportive care (e.g., antipyretics, as required). Clinical decision-making incorporated mNGS results together with clinical presentation, laboratory data, imaging, and history. By 24 h post-*t*_0_, 29.7% of patients with HSIF versus 25.0% of patients with HSTF had defervesced (*p* = 1.000); by 48 h, the proportions were 73.0% versus 37.5% (*p* = 0.029); by 72 h, 78.4% versus 56.3% (*p* = 0.182); and by 96 h, 89.2% versus 68.8% (*p* = 0.027) (**Table 4**). Consistent with these landmark findings, Kaplan–Meier curves favored HSIF (log-rank *p* = 0.025), with an unadjusted HR for defervescence of 2.08 (95% CI, 1.09–3.97). The 6-day cumulative defervescence was 91.9% in HSIF versus 68.8% in HSTF, and by day 12, HSTF reached 93.8%, whereas HSIF was not estimable because all events occurred earlier (**Figure 3**). Taken together, these data support the clinical utility of plasma mNGS not only for etiological clarification but also for therapeutic stratification in patients with nonneutropenic, CMT-negative febrile lung cancer.

## 4 Discussion

### 4.1 Rationale for focusing on the nonneutropenic, CMT-negative population

At our center, routine prophylactic G-CSF administration indicates that truly neutropenic cases, especially febrile neutropenia (FN), are rare. FN also differs fundamentally from the nonneutropenic setting in terms of pathogen spectrum, inflammatory kinetics, and clinical pathways (e.g., immediate empiric broad-spectrum antibiotics are standard). Therefore, we focused on nonneutropenic patients with negative CMT (CMT-negative) to enhance internal validity and interpretability. The tradeoff is limited generalizability: conclusions primarily apply to patients with nonneutropenic lung cancer with fever. In FN, thresholds and dynamics for CRP/PCT and mNGS sampling windows and attribution frameworks require independent validation.

### 4.2 Infectious fever in lung cancer and the role of mNGS

Infectious fever remains the most common complication in oncological practice and can progress to sepsis, organ dysfunction, and death when inadequately controlled (Bhat et al., 2021). Triggers include post-treatment immunosuppression, invasive procedures, and translocation from colonizing niches, including respiratory, gastrointestinal, and bloodstream infections (Yusuf et al., 2023). Conventional diagnostics (cultures and serology) are foundational but limited by modest sensitivity, prolonged turnaround, and narrow coverage, particularly after prior antibiotics (Zhou et al., 2019; Wright et al., 2022; Zhang et al., 2022). In our CMT-negative cohort, plasma mNGS identified at least one organism in 69.8% of the patients, delineating a spectrum that included fastidious bacteria, opportunistic fungi, and herpesviruses. These findings have clinical implications: mNGS-positive patients had higher inflammatory marker levels at presentation and frequently underwent targeted antimicrobial adjustments, whereas mNGS-negative patients often defervesced without antibiotic escalation while continuing oncological or supportive care. Taken together, these observations support the use of plasma mNGS to shorten the empirical, trial-and-error management of diagnostically unresolved fever.

### 4.3 Tumor fever as a diagnosis of exclusion and how mNGS separates signals from noise

TF is a diagnosis of exclusion under major oncology guidance and is difficult to distinguish from infection when early clinical cues overlap (Pasikhova et al., 2017). In this nonneutropenic, CMT-negative subgroup, mNGS functioned as a rapid adjunct to refine attribution. By 48 h after the report, 73.0% of mNGS-positive patients had decreased fever compared with 37.5% of mNGS-negative patients; by 96 h, the proportions were 89.2% and 68.8%, respectively. Although these are unadjusted fixed-time comparisons, the findings are aligned with the intended clinical use of plasma mNGS—prompt, result-guided therapy when infection is likely and avoidance of unnecessary escalation when results are negative—thereby reducing antibiotic exposure while safeguarding patients with true infection.

### 4.4 Positioning within the mNGS literature and implications for practice

In prospective and retrospective studies, mNGS has been associated with a higher diagnostic yield than conventional testing and antimicrobial stewardship actions, including escalation and de-escalation, when appropriate (Wang et al., 2020; Zhou et al., 2021; Xiao et al., 2023). Our study extends this evidence to a precisely defined population of patients with nonneutropenic, CMT-negative lung cancer with fever, where the clinical dilemma is greatest. By restricting the study to diagnostically unresolved cases rather than CMT-positive episodes, we targeted the zone of maximal decision uncertainty and showed that plasma mNGS could inform immediate management, while supporting earlier defervescence. As mNGS diffuses into routine pathways, hybrid strategies that pair plasma mNGS with rapid targeted assays may balance TAT with breadth and can be tailored to local epidemiology and resource environments.

### 4.5 Limitations

This retrospective, single-center analysis with a modest sample size may introduce selection bias and insufficient statistical power, limiting generalizability because an independent validation cohort was not available. First, group assignment is tied to mNGS availability at the report time (*t*_0_), and positive results prompted immediate therapy modification; this design introduces potential confounding by indication and guarantee-time bias in favor of faster symptom control in the mNGS-positive stratum. Second, antipyretics were permitted under routine care; thus, “defervescence” reflects symptom control rather than definitive pathogen eradication, and the confounding effect of antipyretics cannot be fully ruled out. Third, the interpretation of mNGS results relies on clinical adjudication rather than an independent “gold standard” (e.g., histopathology). mNGS carries an inherent risk of detecting incidental findings; low-level viral reads may reflect reactivation or contamination rather than active infection (Wang et al., 2024). Conversely, a single negative mNGS result does not exclude infection when the pathogen burden is low, infection is compartmentalized, or sampling is suboptimal; antibiotic de-escalation based on a negative result should be considered only in clinically stable patients and in conjunction with alternative etiological evidence (Meng et al., 2025). Fourth, mNGS provides limited susceptibility information and, therefore, cannot replace conventional microbiology for antimicrobial guidance (Liu et al., 2024). Our conventional testing panel (blood cultures plus a limited viral serology set) may have underestimated the potential yield of CMT and inflated the comparative advantages of mNGS, especially for viral detection. Finally, although cost, access, and TAT constraints remain barriers, the feasibility of mNGS lies in its targeted application. While currently too costly for routine universal screening, its deployment as a second-line diagnostic tool for clinically difficult cases may offset costs by reducing prolonged hospitalization. Assays are often centralized and may not be reimbursed in some systems, highlighting the need for health-economic evaluations in future studies.

### 4.6 Future directions

Broader integration of plasma mNGS for unexplained febrile illness will require prospective, multicenter studies to validate the standardized reporting criteria. Prospective, multicenter studies should start follow-up at fever onset (rather than *t*_0_), prespecify management pathways, and incorporate time-dependent exposure models that treat mNGS results as time-varying covariates. Blinded, tiered attribution (causative/probable/bystander/contaminant) and read-count thresholds should be harmonized with sensitivity analyses of low-abundance viruses. The key secondary endpoints should include antipyretic-free defervescence, fever burden (temperature area under the curve), time to appropriate therapy, downstream outcomes (intensive care unit transfer, length of stay, mortality), TAT, and cost-effectiveness (especially in resource-limited settings). Negative results should be explicitly evaluated for stewardship value (de-escalation, avoidance of unnecessary diagnostics).

## 5 Conclusion

Plasma mNGS serves as a complementary diagnostic tool in patients with nonneutropenic CMT-negative lung cancer with fever. By substantially increasing pathogen detection in this diagnostically challenging subgroup, mNGS supports etiological clarification and immediate result-guided treatment decisions. Positive results facilitate targeted antimicrobial therapy, whereas negative results, interpreted in a clinical context, support the continuation of oncological or supportive care without unnecessary escalation. This approach enables individualized fever management, helping minimize avoidable antibiotic exposure while preserving timely therapy for true infections. With ongoing technical refinement and prospective validation, plasma mNGS is expected to play an expanding role in diagnostic workflows for complex or uncertain febrile presentations.

## Data Availability

The original contributions presented in the study are included in the article/**Supplementary Material**. Further inquiries can be directed to the corresponding authors.

## References

[B1] Akinosoglou K. S. Karkoulias K. Marangos M. (2013). Infectious complications in patients with lung cancer. Eur. Rev. Med. Pharmacol. Sci. 17, 8–18. 23329518

[B2] Bhat S. Muthunatarajan S. Mulki S. S. Bhat K. A. Kotian K. H. (2021). Bacterial infection among cancer patients: Analysis of isolates and antibiotic sensitivity pattern. Int. J. Microbiol. 2021, 8883700. doi: 10.1155/2021/8883700. PMID: 33510793 PMC7825358

[B3] Clinical and Laboratory Standards Institute (CLSI) . (2023). Performance standards for antimicrobial susceptibility testing (Wayne, PA: Clinical and Laboratory Standards Institute).

[B4] Diao Z. Han D. Zhang R. Li J. (2022). Metagenomics next-generation sequencing tests take the stage in the diagnosis of lower respiratory tract infections. J. Adv. Res. 38, 201–212. doi: 10.1016/j.jare.2021.09.012. PMID: 35572406 PMC9091713

[B5] Goldberg B. Sichtig H. Geyer C. Ledeboer N. Weinstock G. M. (2015). Making the leap from research laboratory to clinic: Challenges and opportunities for next-generation sequencing in infectious disease diagnostics. mBio 6, e01888-01815. doi: 10.1128/mBio.01888-15. PMID: 26646014 PMC4669390

[B6] Grumaz S. Stevens P. Grumaz C. Decker S. O. Weigand M. A. Hofer S. . (2016). Next-generation sequencing diagnostics of bacteremia in septic patients. Genome Med. 8, 73. doi: 10.1186/s13073-016-0326-8. PMID: 27368373 PMC4930583

[B7] Gu W. Miller S. Chiu C. Y. (2019). Clinical metagenomic next-generation sequencing for pathogen detection. Annu. Rev. Pathol. 14, 319–338. doi: 10.1146/annurev-pathmechdis-012418-012751. PMID: 30355154 PMC6345613

[B8] Han D. Li Z. Li R. Tan P. Zhang R. Li J. (2019). mNGS in clinical microbiology laboratories: On the road to maturity. Crit. Rev. Microbiol. 45, 668–685. doi: 10.1080/1040841x.2019.1681933. PMID: 31691607

[B9] Li H. Durbin R. (2009). Fast and accurate short read alignment with Burrows-Wheeler transform. Bioinformatics 25, 1754–1760. doi: 10.1093/bioinformatics/btp324. PMID: 19451168 PMC2705234

[B10] Lin P. Chen Y. Su S. Nan W. Zhou L. Zhou Y. . (2022). Diagnostic value of metagenomic next-generation sequencing of bronchoalveolar lavage fluid for the diagnosis of suspected pneumonia in immunocompromised patients. BMC Infect. Dis. 22, 416. doi: 10.1186/s12879-022-07381-8. PMID: 35488253 PMC9052728

[B11] Liu Y. Qin S. Lan C. Huang Q. Zhang P. Cao W. (2024). Effectiveness of metagenomic next-generation sequencing in the diagnosis of infectious diseases: A systematic review and meta-analysis. Int. J. Infect. Dis. 142, 106996. doi: 10.1016/j.ijid.2024.106996. PMID: 38458421

[B12] Marik P. E. (2000). Fever in the ICU. Chest 117, 855–869. doi: 10.1378/chest.117.3.855. PMID: 10713016

[B13] Meng B. Liu H. Wu Q. Qu L. Mao C. Yang F. . (2025). Antimicrobial strategies of lower respiratory tract infections in immunocompromised patients based on metagenomic next-generation sequencing: A retrospective study. BMC Infect. Dis. 25, 360. doi: 10.1186/s12879-025-10753-5. PMID: 40087607 PMC11907972

[B14] Messacar K. Parker S. K. Todd J. K. Dominguez S. R. (2017). Implementation of rapid molecular infectious disease diagnostics: The role of diagnostic and antimicrobial stewardship. J. Clin. Microbiol. 55, 715–723. doi: 10.1128/jcm.02264-16. PMID: 28031432 PMC5328439

[B15] Miao Q. Ma Y. Wang Q. Pan J. Zhang Y. Jin W. . (2018). Microbiological diagnostic performance of metagenomic next-generation sequencing when applied to clinical practice. Clin. Infect. Dis. 67, S231–S240. doi: 10.1093/cid/ciy693. PMID: 30423048

[B16] Pasikhova Y. Ludlow S. Baluch A. (2017). Fever in patients with cancer. Cancer Control 24, 193–197. doi: 10.1177/107327481702400212. PMID: 28441374

[B17] Reyna-Figueroa J. Lagunas-Martínez A. Martínez Matsumoto P. Madrid-Marina V. (2015). Procalcitonin as a diagnostic biomarker of sepsis in children with cancer, fever and neutropenia: Literature review. Arch. Argent. Pediatr. 113, 46–52. doi: 10.5546/aap.2015.46. PMID: 25622161

[B18] Rodino K. G. Simner P. J. (2024). Status check: Next-generation sequencing for infectious-disease diagnostics. J. Clin. Invest. 134, 1–4. doi: 10.1172/jci178003. PMID: 38357923 PMC10866643

[B19] Rolston K. V. (2017). Infections in cancer patients with solid tumors: A review. Infect. Dis. Ther. 6, 69–83. doi: 10.1007/s40121-017-0146-1. PMID: 28160269 PMC5336421

[B20] Rosolem M. M. Rabello L. S. Lisboa T. Caruso P. Costa R. T. Leal J. V. . (2012). Critically ill patients with cancer and sepsis: Clinical course and prognostic factors. J. Crit. Care 27, 301–307. doi: 10.1016/j.jcrc.2011.06.014. PMID: 21855281

[B21] Sahoo M. K. Lefterova M. I. Yamamoto F. Waggoner J. J. Chou S. Holmes S. P. . (2013). Detection of cytomegalovirus drug resistance mutations by next-generation sequencing. J. Clin. Microbiol. 51, 3700–3710. doi: 10.1128/jcm.01605-13. PMID: 23985916 PMC3889754

[B22] Salipante S. J. Hoogestraat D. R. Abbott A. N. SenGupta D. J. Cummings L. A. Butler-Wu S. M. . (2014). Coinfection of Fusobacterium nucleatum and Actinomyces Israelii in mastoiditis diagnosed by next-generation DNA sequencing. J. Clin. Microbiol. 52, 1789–1792. doi: 10.1128/jcm.03133-13. PMID: 24574281 PMC3993667

[B23] Shomali W. Hachem R. Chaftari A. M. Jiang Y. Bahu R. Jabbour J. . (2012). Can procalcitonin distinguish infectious fever from tumor-related fever in non-neutropenic cancer patients? Cancer 118, 5823–5829. doi: 10.1002/cncr.27602. PMID: 22605389

[B24] Sung H. Ferlay J. Siegel R. L. Laversanne M. Soerjomataram I. Jemal A. . (2021). Global cancer statistics 2020: GLOBOCAN estimates of incidence and mortality worldwide for 36 cancers in 185 countries. CA Cancer J. Clin. 71, 209–249. doi: 10.3322/caac.21660. PMID: 33538338

[B25] Tang W. Zhang Y. Luo C. Zhou L. Zhang Z. Tang X. . (2021). Clinical application of metagenomic next-generation sequencing for suspected infections in patients with primary immunodeficiency disease. Front. Immunol. 12. doi: 10.3389/fimmu.2021.696403. PMID: 34484193 PMC8414648

[B26] Wang C. Yin X. Ma W. Zhao L. Wu X. Ma N. . (2024). Clinical application of bronchoalveolar lavage fluid metagenomics next-generation sequencing in cancer patients with severe pneumonia. Respir. Res. 25, 68. doi: 10.1186/s12931-023-02654-5. PMID: 38317206 PMC10840150

[B27] Wang J. Han Y. Feng J. (2019). Metagenomic next-generation sequencing for mixed pulmonary infection diagnosis. BMC Pulm. Med. 19, 252. doi: 10.1186/s12890-019-1022-4. PMID: 31856779 PMC6921575

[B28] Wang S. Ai J. Cui P. Zhu Y. Wu H. Zhang W. (2020). Diagnostic value and clinical application of next-generation sequencing for infections in immunosuppressed patients with corticosteroid therapy. Ann. Transl. Med. 8, 227. doi: 10.21037/atm.2020.01.30. PMID: 32309374 PMC7154484

[B29] Wooley J. C. Godzik A. Friedberg I. (2010). A primer on metagenomics. PloS Comput. Biol. 6, e1000667. doi: 10.1371/journal.pcbi.1000667. PMID: 20195499 PMC2829047

[B30] Wright W. F. Simner P. J. Carroll K. C. Auwaerter P. G. (2022). Progress report: Next-generation sequencing, multiplex polymerase chain reaction, and broad-range molecular assays as diagnostic tools for fever of unknown origin investigations in adults. Clin. Infect. Dis. 74, 924–932. doi: 10.1093/cid/ciab155. PMID: 33606012

[B31] Xiao Y. H. Liu M. F. Wu H. Xu D. R. Zhao R. (2023). Clinical efficacy and diagnostic value of metagenomic next-generation sequencing for pathogen detection in patients with suspected infectious diseases: A retrospective study from a large tertiary hospital. Infect. Drug Resist. 16, 1815–1828. doi: 10.2147/idr.S401707. PMID: 37016633 PMC10066896

[B32] Yusuf K. Sampath V. Umar S. (2023). Bacterial infections and cancer: Exploring this association and its implications for cancer patients. Int. J. Mol. Sci. 24, 3110. doi: 10.3390/ijms24043110. PMID: 36834525 PMC9958598

[B33] Zell J. A. Chang J. C. (2005). Neoplastic fever: A neglected paraneoplastic syndrome. Support Care Cancer 13, 870–877. doi: 10.1007/s00520-005-0825-4. PMID: 15864658

[B34] Zhang X. Li Y. Yin J. Xi B. Wang N. Zhang Y. (2022). Application of next-generation sequencing in infections after allogeneic haematopoietic stem cell transplantation: A retrospective study. Front. Cell. Infect. Microbiol. 12. doi: 10.3389/fcimb.2022.888398. PMID: 35774403 PMC9239075

[B35] Zhao Z. Li X. Zhao Y. Wang D. Li Y. Liu L. . (2018). Role of C-reactive protein and procalcitonin in discriminating between infectious fever and tumor fever in non-neutropenic lung cancer patients. Med. (Baltimore) 97, e11930. doi: 10.1097/md.0000000000011930. PMID: 30113495 PMC6112972

[B36] Zhou H. Larkin P. M. K. Zhao D. Ma Q. Yao Y. Wu X. . (2021). Clinical impact of metagenomic next-generation sequencing of bronchoalveolar lavage in the diagnosis and management of pneumonia: A multicenter prospective observational study. J. Mol. Diagn. 23, 1259–1268. doi: 10.1016/j.jmoldx.2021.06.007. PMID: 34197923

[B37] Zhou X. Wu H. Ruan Q. Jiang N. Chen X. Shen Y. . (2019). Clinical evaluation of diagnosis efficacy of active Mycobacterium tuberculosis complex infection via metagenomic next-generation sequencing of direct clinical samples. Front. Cell. Infect. Microbiol. 9. doi: 10.3389/fcimb.2019.00351. PMID: 31681628 PMC6813183

